# *In Vitro* Selection of Cathepsin E-Activity-Enhancing Peptide Aptamers at Neutral pH

**DOI:** 10.1155/2011/834525

**Published:** 2011-03-22

**Authors:** Madhu Biyani, Masae Futakami, Koichiro Kitamura, Tomoyo Kawakubo, Miho Suzuki, Kenji Yamamoto, Koichi Nishigaki

**Affiliations:** ^1^Department of Functional Materials Science, Graduate School of Science and Engineering, Saitama University, 255 Shimo-Okubo, Saitama 338-8570, Japan; ^2^City Area Program Saitama Metropolitan Area, Saitama Small and Medium Enterprises Development Corporation, 2-3-2 Kamiochiai, Chuo-Ku, Saitama 338-0001, Japan; ^3^Rational Evolutionary Design of Advanced Biomolecules, Saitama (REDS) Group, Saitama Small Enterprise Promotion Corporation, no. 552 Saitama Industrial Technology Center, 3-12-18 Kami-Aoki, Kawaguchi, Saitama 333-0844, Japan; ^4^Janusys Corporation, no. 508 Saitama Industrial Technology Center, 3-12-18 Kami-Aoki, Kawaguchi, Saitama 333-0844, Japan; ^5^Proteolysis Research Laboratory, Graduate School of Pharmaceutical Sciences, Kyushu University, Higashi-Ku, Fukuoka 812-8582, Japan

## Abstract

The aspartic protease cathepsin E has been shown to induce apoptosis in cancer cells under physiological conditions. Therefore, cathepsin E-activity-enhancing peptides functioning in the physiological pH range are valuable potential cancer therapeutic candidates. Here, we have used a general *in vitro* selection method (evolutionary rapid panning analysis system (eRAPANSY)), based on inverse substrate-function link (SF-link) selection to successfully identify cathepsin E-activity-enhancing peptide aptamers at neutral pH. A successive enrichment of peptide activators was attained in the course of selection. One such peptide activated cathepsin E up to 260%, had a high affinity (K_D_; *∼*300 nM), and had physiological activity as demonstrated by its apoptosis-inducing reaction in cancerous cells. This method is expected to be widely applicable for the identification of protease-activity-enhancing peptide aptamers.

## 1. Introduction

Besides other targets like kinases and membrane receptors, proteases are one of the most effective targets for drug discovery [1, 2]. To date, protease-inhibitors have been widely developed whereas protease-activators, which have great potential in medical and scientific uses [3, 4], have been far less developed due to technical difficulties [5]. These include the fact that the activation mechanism, which is usually regulated by the binding of ligands, that is, allosteric effectors, rather than by substrates [6], is very difficult to predict in detail from theory. In contrast, the inhibition mechanism can be principally assigned to competitive binding of inhibitors to the active site or steric hindrance of the normal action of enzyme. The latter can be inferred from the molecular structure at atomic resolution [7, 8]. 

 To address the above difficulty, we have recently developed a systemic *in vitro* evolution method (called eRAPANSY (evolutionary rapid panning analysis system)) [9] for screening of protease-regulating peptide aptamers based on mRNA (and cDNA) display [10–12] and a substrate-function link (SF-link) method [13]. This method was described in the successful identification of cathepsin E- (CatE-) inhibitory peptide aptamers [9]. As a next step, we attempted to obtain CatE-activity-enhancing peptide aptamers at neutral pH (pH 7.4) using this method since CatE was reported to induce apoptosis of cancer cells under physiological conditions (pH 7.4). In this process, CatE catalyzes the release of TRAIL (tumor necrosis factor-related apoptosis-inducing ligand) without affecting normal cells [14]. Thus, CatE-activity-enhancing peptide aptamers that function at neutral pH may be promising reagents for cancer therapeutics. Intriguingly, a similar approach that utilizes a microenvironmentally activated protease has been developed, and clinical trials using protease-activated prodrugs for prostate cancer are path-finding approaches in this field [15]. 

In order to identify CatE-activity-enhancing peptide aptamers at neutral pH, we first developed an assay system for measuring the unusual activity of cathepsin E at pH 7.4 instead of pH 4.5 and then performed the general scheme of *in vitro* evolution (eRAPANSY) for obtaining protease-activating peptides based on inverse SF-link selection. We describe here the successful development of one promising peptide aptamer (S_3_) that enhanced CatE-activity by up to 260% *in vitro* and which induced apoptosis in cancer cells (HeLa). Through these experiments, we have demonstrated and confirmed the effectiveness of this method for *in vitro* evolution and its wide applicability.

## 2. Materials and Methods

### 2.1. Cathepsin E (CatE) and Its Substrate

A fluorogenic substrate susceptible at neutral pH to the protease cathepsin E, which usually functions at acidic pH, was designed as per reference [16]: [Nma-Gly-Gly-Arg-Arg-Ser-Gly-Thy-Cys-Gly(Dnp)-D-Arg-NH_2_]. The peptide was custom-synthesized at Peptide Institute (Osaka, Japan) and confirmed to be an effective substrate at pH 7.4. The peptide was dissolved in dimethyl sulfoxide (DMSO) at 10 mM and stored in a deep freezer until use. The enzyme CatE was prepared from rat spleen according to the method previously described [17] and immobilized on *N*-hydroxysuccinimide-activated sepharose beads (GE Healthcare, USA) using the amine-coupling method and stored in, 100 mM phosphate buffer (pH 7.4) at 4°C.

### 2.2. CatE-Activity-Enhancement Assay

CatE-activation by peptide aptamers was assayed by the method described previously [9] but with a few modifications. Briefly, the CatE (20 nM) solution preincubated with a peptide aptamer in a 1 : 1 molar ratio in the selection buffer (50 mM Tris-HCl, 100 mM NaCl, pH 7.4) at 25°C for 10 min. was combined with the fluorogenic substrate (5 *μ*M) and the enzyme reaction performed at 37°C for 1 h. The reaction product was measured by fluorescence intensity at 440 nm with an excitation wavelength of 340 nm using a fluorescence microplate reader, Infinite 200 (Tecan, Japan). The percentage of activation (*A*) was calculated by fitting the data to the following equation:

(1) $$A=100\times \frac{{\text{S}}_{f}-B_{f}}{C_{f}-B_{f}}\;\left[\%\;\text{activation}\right],$$

where S_*f*_ represents the fluorescence intensity of the positive reaction consisting of the enzyme (CatE), the fluorogenic substrate, and a possible peptide activator; *C*_*f*_ is that of the control reaction mixture consisting of CatE and fluorogenic substrate (without peptide activator); *B*_*f*_ is the background fluorescence of the negative reaction mixture consisting of only the fluorogenic substrate.

### 2.3. Primary and Secondary Library Constructions

**Figure 1:**
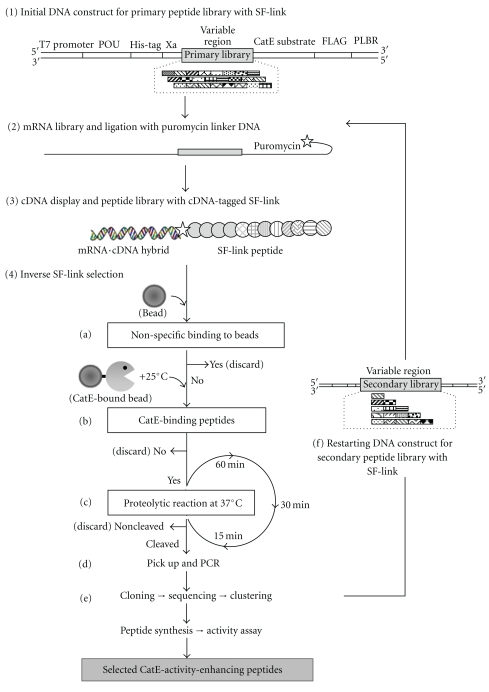
Schematic representation of the inverse SF-link selection method for screening of CatE-activity-enhancing peptides at neutral pH. A DNA template construct for primary peptide library with SF-link is shown where YLBS-generated variable region is integrated with T7 promoter, POU-domain, His-tag, Factor Xa sequences at upstream part, and further elongated with CatE substrate, FLAG, and PLBR (Puromycin linker binding region) sequences at downstream part (step 1). DNA template library is transcribed into mRNA library followed by ligation with Puromycin-linker DNA at the 3′-terminus end (step 2) and then converted into cDNA-displayed SF-link peptide products which is composed of both the functional part (random peptide sequence) and CatE peptide substrate (step 3). Finally, inverse SF-link selection (step 4) was performed using cDNA-tagged SF-link peptide library: (a) the nonspecific binding peptides were removed by *NHS-*activated sepharose beads. (b) The CatE binding peptides were recovered at 25°C after 10 min. of incubation with CatE-bound beads. (c) CatE-binding peptides were subjected to protease (CatE) cleavage at 37°C with cutoff incubation time in three successive rounds. (d) The rapidly cleaved peptides (still attached to cDNA) were recovered, PCR-treated, and used for the next selection round. (e) Molecules selected were subjected to cloning, sequencing, and clustering analysis. Some of the selected clones were peptide synthesized [18], analyzed for activity, and confirmed as CatE-activity-enhancing peptides. (f) The secondary library was generated using the primary library selection products followed by the peptide library with cDNA-tagged SF-link generation and selection process as described in steps 2–4.

**Figure 2:**
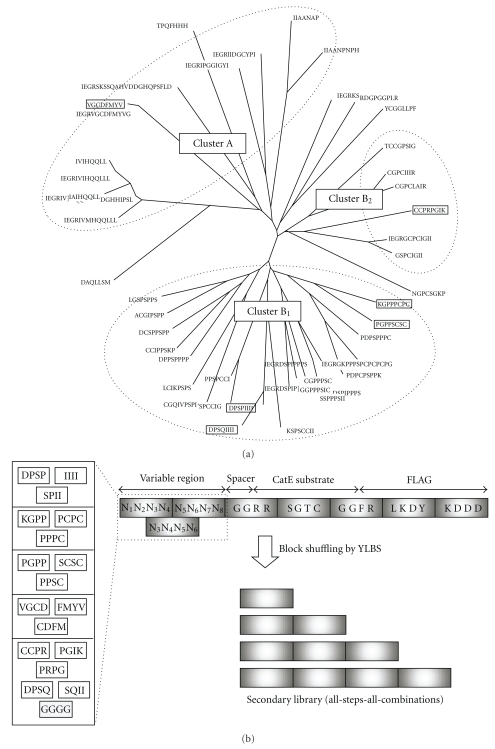
Surveying the building block peptides used for the secondary library. (a) Cluster analysis of the peptides selected from the primary library, using ClustalW supported by DDBJ (DNA Data Bank of Japan). Consensus sequences are shown in bold. (b) Schematic drawing of 23 tetramer peptide blocks used for the secondary library construction by the ASAC (all-steps-all-combinations) method. In addition to the blocks from the selected sequences (which also contain the SF-link construct), arbitrarily chosen blocks of (GGGG)/*n* were combined.

The primary and the secondary libraries were constructed according to the protocols described in [9]. In brief, the primary library was constructed by Y-ligation-based block shuffling (YLBS) [19]. The secondary library was constructed by exploiting the YLBS method with slight modifications. The peptide sequences obtained from the primary library selection were cluster-analyzed and used to design the subsequently constructed blocks (Figure 2). Using these blocks, YLBS-shuffling was performed (Table 1). The resultant library contained all of the arbitrarily shuffled blocks with a different number of blocks (1–4 blocks; Figure 2(b)), thus termed an all-steps-all-combinations (ASAC) library. Prior to the *in vitro* selection, the variable sequences were integrated into the cDNA-tagged-SF-link peptide construct. The preparation of the construct was performed following the method previously reported [9] and is partially appearing in Figure 1. 

### 2.4. Selection of CatE-Activity-Enhancing Peptide Aptamers

The cDNA displayed-SF-link construct having an intervening CatE-substrate sequence (Figure 1) was subjected to the inverse SF-link selection method. An inverse function-based selection strategy was adopted to obtain CatE-activating peptides at neutral pH (pH 7.4) and is described below. First, in order to remove the nonspecific binding peptides, the cDNA-tagged SF-link-peptide library (see Figure 1) was treated with the selection buffer (50 mM Tris-HCl, 100 mM NaCl, 5 mM MgCl_2_, pH 7.4) containing 5 *μ*L of *NHS*-activated sepharose beads (90 *μ*m mean particle diameter, resuspended in 100% isopropanol) at 25°C for 10 min. The supernatant containing unbound cDNA-tagged-SF-link peptide molecules was collected after removing the beads by spinning down and used in the succeeding experiment. The cDNA-tagged-SF-link peptide library thus obtained was incubated with CatE-immobilized beads (about 5 pmol CatE immobilized) at 25°C for 10 min. Those peptides which bind to beads (in other words, peptides with affinity for CatE), were collected by spinning and immediately resuspended in reaction buffer (50 mM Tris-HCl, 1 M NaCl, pH 7.4) and incubated at 37°C for 1 h. (1st Round), 30 min. (2nd Round), or 15 min. (3rd Round). The incubation time decreases as the selection round progresses leading to enrichment of rapidly cleaved species. The reaction was terminated by adding 1 mL of 5% trichloroacetic acid. The supernatant expected to contain CatE-activators was collected, PCR-treated, and used as the library for the next selection round. The molecules thus selected were subjected to conventional cloning, sequencing analyses, synthesis, and activity assay.

### 2.5. In Vitro Translation Synthesis and Chemical Synthesis of Peptides

For the rapid synthesis of peptides, *in vitro *translation using the coding sequences of selected peptides was used [18]. The DNA construct consisting of the sequences for the relevant peptide, the streptavidin-binding peptide, and the protease factor Xa recognition site was prepared by way of PCR and then transcribed and finally translated *in vitro *according to the method previously reported in [18]. 

Those peptides which were cathepsin E-activating in these experiments were subjected to commercial chemical synthesis (BEX Corporation Ltd., Tokyo, Japan; SCRUM Corporation Ltd., Tokyo, Japan). These peptides were purified by HPLC and confirmed by MALDI-TOF-MS.

### 2.6. Measurement of K_D_

An SPR instrument, Biacore 2000 (GE Healthcare, UK), was used for measuring K_D_ for the binding of cathepsin E and selected peptides. Cathepsin E was immobilized onto Biacore sensor chip CM5 (GE Healthcare, UK) by the amine-coupling method using an Amine Coupling Kit (GE Healthcare, UK). Immobilization of cathepsin E was performed using running buffer (50 mM sodium acetate, 100 mM NaCl, pH 4.5). To determine dissociation constants, four different concentrations of selected peptides were used for the interaction. The peptide interactions were performed against CatE using the running buffer (50 mM Tris-HCl, 100 mM NaCl, pH 7.4). After each measurement, the chip surface was regenerated with 50 mM NaOH. The binding data were analyzed with the 1 : 1 binding with mass transfer model in the BIAevaluation software version 4 : 1 (Biacore).

### 2.7. Measurement of Cell Viability

HeLa cells (1 × 10^4^ cells/well in 96-well plate) were treated with cathepsin E (7.7 *μ*M) alone and also in addition with peptide aptamer (P_3_, selected from the primary library) using three different molar ratios of cathepsin E: peptide aptamer = 1 : 10; 1 : 100; 1 : 1000 in 100 *μ*L of serum-free Opti-MEM at 37°C for 20 h. The cells' viability was measured with the use of a Cell Counting Kit-8 (Dojindo Molecular Technologies, Japan; Figure 4(a)). The same method was also used for measuring the effect of cathepsin E and peptide aptamer (S_3_, selected from the secondary library) on HeLa cells. The cell viability was measured with Cell Counting Kit-F (Dojindo Molecular Technologies, Japan; Figure 4(b)).

### 2.8. Statistics

All data were expressed as mean ± SEMs. For statistical analysis, two-way analysis of variance (Student's *t*-test) with repeated measures was used. *P* < .05 was taken as the level of statistical significance.

## 3. Results

### 3.1. A General Scheme of In Vitro Evolution Method Based on Inverse SF-Link Selection

To identify CatE-activity-enhancing peptides at neutral pH, we adopted an *in vitro* evolution method (eRAPANSY) [9] based on the inverse SF-link selection approach which is shown schematically in Figure 1. In brief, the overall strategy consists of the primary library construction and selection (mass selection and clonal selection) followed by construction and enrichment through selection of a secondary library (a peptide library generated by block shuffling of all-step-all-combinations using the primary library selection products). 

In the mass selection (i.e., dealing with a population of molecules as a whole), the variable sequences (possible “functional” sequences) were integrated into the initial DNA construct flanked by the substrate peptide sequence (step 1 in Figure 1). Namely, each peptide was attached to the DNA molecule which consisted of coding regions for T7 promoter, Kozak, His-tag, Factor Xa recognition sequence, variable sequence, CatE substrate sequence, antigenic sequence for the FLAG antibody, and puromycin linker binding region (PLBR; Figure 1). The cDNA display method [12], a derivative of mRNA display [10, 11], was applied to this DNA library to obtain the peptide library with the cDNA-tagged SF-link. To prepare the cDNA display construct, a puromycin linker DNA was bound to an mRNA molecule through the hybridization with the PLBR sequence (step 2 in Figure 1). The puromycin-linked mRNA thus formed can covalently bind to the nascent peptide via the puromycin, a mimic of an aminoacyl tRNA, generating the mRNA display construct. To increase the stability, the mRNA display construct was converted to the cDNA display one by the action of reverse-transcriptase. The peptide library with the cDNA-tagged SF-link construct thus obtained contains a random peptide (functional part) and a CatE-substrate sequence [9] (step 3 in Figure 1) and was subjected to the inverse SF-link selection (step 4a–4d, for details see Materials and Methods) devised for screening of the CatE-activity-enhancing peptide aptamers. The essential difference between the SF-link selection method (to identify inhibitors [9]) and the inverse SF-link selection method (to identify activators) is that the noncleaved peptides were discarded while the cleaved peptides were retained as shown in step 4c. 

In the clonal selection (i.e., screening of the molecules individually on a clone-by-clone basis), the peptide aptamers thus obtained from mass selection were cloned, sequenced, and cluster analyzed (step 4e). Some of clones were subjected to *in vitro* peptide translation [18] and the CatE-enhancing activity confirmed in an activity assay as described in the Materials and Methods. After the primary library generation and selection, the secondary library generation and selection results in a proven elevation in the activity of selected CatE-peptide activators (step 4f).

### 3.2. CatE-Activity-Enhancing Peptide Aptamers Selected from the Primary and the Secondary Library

**Figure 3:**
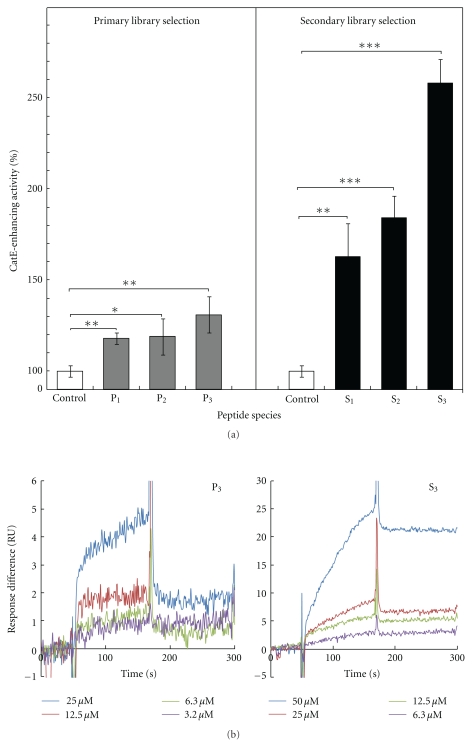
Selection of cathepsin E-activity-enhancing peptides at neutral pH. (a) Selection products from the primary library and the secondary library. For both, the top 3 peptides are shown. For the activity assay, the peptides used here were synthesized by an *in vitro *translation method [18]. Here, the ratio of CatE to an activity-enhancing peptide was 1/1 (= 20 nM/20 nM (see Materials and Methods for detail). Each error bar represent average and standard deviation values of three independent experiments. (b) Biacore sensorgrams of peptides, P_3_ (primary library selection product) and S_3_ (secondary library one) against cathepsin E (3.36 mg/mL) immobilized onto sensor chip CM5. To determine dissociation constants, four different concentrations (25, 12.5, 6.3, and 3.2 *μ*M for P_3_ and 50, 25, 12.5, and 6.3 *μ*M for S_3_) of the peptides were subjected to the interaction. *P < .08, **P < .05, and ***P < .0003 compared with the control group CatE and substrate only (without peptide activator), by Student's *t*-test.

The peptide aptamers thus obtained from the primary library (P_1_, P_2,_ and P_3_) enhanced the CatE-activity by around 10 to 30% (measured using peptides obtained by *in vitro* translation synthesis) and such activities were also confirmed by using chemical synthesis of peptides (Table 2(A)). Notably, the peptide with the highest activity in the primary library selection was an insertion-carrying peptide (P_3_) of 13 amino acids while others are of normal size (octapeptide). The resulting CatE-activity-enhancing peptide aptamers thus obtained from the primary library were cluster-analyzed to provide sequence information on possible functional blocks which could be used for generating the secondary library (ASAC library; Table 1; and Figure 2(a)). The secondary library was constructed as shown in Figure 2(b) [9]. The secondary cDNA-tagged SF-link peptide library thus constructed was subjected to the inverse SF-link selection as described above (Figure 1). The selection products from this library demonstrated higher activity than those selected from the primary library (Figure 3(a) and Table 2). The average increase in the CatE-enhancing activity of the top three hits selected out of ~50 clones sequence-analyzed was significant with peptides demonstrating more than 160% activity and one peptide (S_3_) showing an activity increase of up to 260% CatE activity (*P* < .0003; Table 2), which is two-fold higher activity than that (130%) of the best peptide (P_3_; *P* < .05) obtained from the primary library selection when measured using *in vitro* translation synthesized peptide (Figure 3(a)). However, such values are known to have a relatively large fluctuation [18] since the amount of the final product (peptide) generated by *in vitro* translation is an estimation value based on the initial amount of the reactant due to their very small n amount (~pmole) [18]. Therefore, peptide (S_3_) was chemically synthesized, and its CatE-enhancing activity was measured (Table 2(B)). The value thus obtained was lower than that obtained from *in vitro* translation, which is a trend generally observed with this approach [18]. As a result, a higher activity (168%) than the best peptide in the primary screen (130%) was confirmed, proving the steady rise in the activity of peptides in this approach. The dissociation constant of the peptide (S_3_) was found to be 3.89 × 10^−7^M by the SPR method, thus showing a relatively strong binding affinity (Table 2(B) and Figure 3(b)). The selection thus performed using the inverse SF-link method was able to identify CatE-activity-enhancing peptide aptamers at neutral pH (Figure 1 and Table 2), indicating the effectiveness of the inverse SF-link selection method. The effectiveness of the secondary library selection is clearly shown in Figure 3.

### 3.3. Evaluation of Biological Activity of Selected Peptide Aptamers in Cells

**Figure 4:**
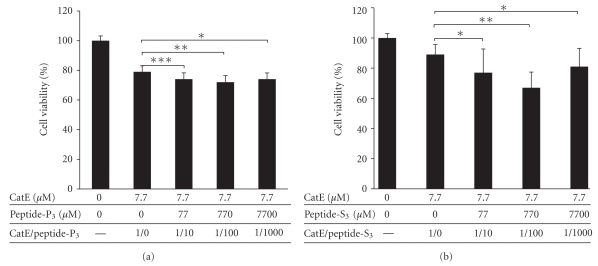
Effect of CatE and peptide aptamers, (a) P_3_  and (b) S_3_, on HeLa cells. HeLa cells (*n* = 1 × 10^4^) in 96-well plates were incubated overnight with, cathepsin E and peptide (P_3_ or S_3_) mixture at three different molar concentration ratios (cathepsin E : peptide = 1 : 10; 1 : 100; 1 : 1000). After 20 hours of incubation at 37°C, cells were assayed for viability using a Cell Counting Kit-8 (measuring the absorbance at 450 nm) and Cell Counting Kit-F (CCK-F) measuring the fluorescence at 535 nm (excitation at 485 nm) using a microplate reader. Error bars represent average and standard deviation values of three independent experiments. *P < .4, **P < .05, and ***P < .007 compared with the control group (only CatE-treated cells) by Student's *t*-test.

In order to evaluate the activities of selected peptides (P_3_ and S_3_ obtained from the primary and the secondary libraries, resp.; Table 2), we next tested the combined effect of CatE and selected peptide aptamers on HeLa cells. The cells were treated with CatE alone or with CatE and peptide aptamers (P_3_ and S_3_) in a dose-dependent manner. Any decrease in cellular viability was analyzed (for details see Material and Methods). Cells treated with CatE and a peptide aptamer, P_3_ or S_3_, at a molar ratio of 1 : 100 showed around a 1.5- and 3-fold increase in the level of apoptosis in comparison to cells treated with CatE alone (*P* < .05; Figure 4), respectively. These positive results corroborate our current approach of using an *in vitro *evolution method modified with the inverse SF-link selection for identifying protease activity-enhancing peptide aptamers.

## 4. Discussion

CatE is reported to have the ability to induce apoptosis in cancer cells [14], a process which must occur under physiological conditions (i.e., at neutral pH). In this study, the activity of CatE, which is known to usually function at acidic pH (pH 4.5), was measured at neutral pH (pH 7.4) using a novel substrate as described in Materials and Methods. The peptides that can enhance CatE activity under the assay conditions were selected as shown in Table 2. In this paper, we address two subjects: (i) selection of activity-enhancing, rather than inhibiting peptides and (ii) performance of evolution, not only selection, of peptides (proteins). Both points are discussed here. It is well known that finding activating reagents is more difficult than finding inhibitory ones since the latter has the clear target of the substrate-binding site. Therefore, a lot of inhibitory reagents have been developed but far fewer activating ones [4, 5, 20, 21]. The SF- (substrate-function-) link method developed recently [13] has the potential to select protease-activating peptides if it is operated in the inverse mode owing to its construction design (see Figure 1), and this technique has been successfully exploited for the first time in this paper. This has resulted in the finding that the majority of selected peptides are CatE-activating species (partly shown in Table 2). This is noteworthy since starting with the same library (the affinity selection product), we could obtain the opposite type of functional peptides (activators) by adopting the inverse mode of function-based selection using the SF-link method in which swiftly cleaved off cDNA-tagged SF-link peptide molecules (which are a conjugate of information and functional molecules, see Figure 1 step 4c, were collected). The cathepsin E-activity-enhancing peptides (P_1_, P_3_, S_2,_ and S_3_) identified herein contain common features being composed of a charged head group and a large hydrophobic tail (detergent-like peptides). The activation may be due to some combination of enzyme stabilization in solution, loosening of the protein secondary structure, or alteration of adsorption of the enzyme to the surface of containers during the enzyme reaction [22]. We need to further investigate these possible activation mechanisms. Interestingly, in the SPR measurements, the secondary library selection resulted in obtaining peptides with increased activity without much change in affinities (similar K_D_ values for P_3_, from the primary library, and S_3_, from the secondary library and see Table 2 and Figure 3(b)). Those peptides (P_1_ and S_2_) which contain a single charged amino acid embedded in hydrophobic ones showed abnormal behaviors in the SPR measurement (data not shown), probably indicating strong nonspecific (hydrophobic) binding to the supporting plate even though a surface-blocking agent (1 M ethanolamine-HCl, pH 8.5) was used. Therefore, these peptides may need to be modified into a more soluble structure for advanced applications such as a drug seed. In addition, the evaluation of selected CatE-activity-enhancing peptides on cells provides definitive proof that our current *in vitro* evolution method based on the inverse SF-link selection is effective.

In addition, this study also confirms the effectiveness of the eRAPANSY method that allows the molecular library to progress towards higher activity. Although it may be early to draw the conclusion that this is a proven method for obtaining much improved molecules, the following can be stated safely: the primary library selection is equivalent to a search for module-like peptides out of a huge number of meaningless peptides while the construction of the secondary library is analogous to module-shuffling (or exon shuffling), which is assumed to have worked during the evolution of proteins [23]. An interesting issue to be addressed in the future concerns the size of blocks (or module) which holds meaning (or function), though, in this study, it was rather arbitrarily determined to be a tetrapeptide, which was ultimately effective. In other words, the way, by which natural proteins evolved, holds promise for the artificial development of proteins. According to recent genome analysis, during the evolution of life, the recombination mechanism strongly governed and contributed to the generation of novel proteins [24, 25]. This fact is also supportive of our approach where drastic recombination of sequences (block shuffling) has been employed together with point mutation in the construction of the secondary library.

 In conclusion, this study has succeeded in introducing an assay system for CatE activity at neutral pH (pH 7.4) and in obtaining CatE-activating peptides by employing the successive *in vitro* evolution method (eRAPANSY) based on inverse SF-link selection. These results endorse the notion that the method employed here is applicable for obtaining activity-enhancing peptides for proteases at large. Such a notion, of course, needs to be strengthened by succeeding studies adopting this method.

## Figures and Tables

**Table 1:** Oligonucleotides used for the secondary library (ASAC) construction for CatE-activating peptides at neutral pH.

No.	Nucleotide sequence (5′→3′)	Size (nucleotides)	Amino acid sequence of the block
*Definition* ^ a^			

	GGCTCGCGAA TACTGCGAAG AAAGGTCGT (D_5_)	29	
	GATCTCACTC CTTCGCAGTA TTCGCGAGCC (D_3_)	30	

*Starting *5′/3′* -halves for ASAC library *			

1	D_5_-GACCCATCCC CA/GACCCATCCC CA-D_3_	41/42	DPSP
2	D_5_-ATTATTATTA TT/ATTATTATTA TT-D_3_	41/42	IIII
3	D_5_-AAAGGCCCAC CA/AAAGGCCCAC CA-D_3_	41/42	KGPP
4	D_5_-CCATGCCCAT GC/CCATGCCCAT GC-D_3_	41/42	PCPC
5	D_5_-CCAGGCCCAC CA/CCAGGCCCAC CA-D_3_	41/42	PGPP
6	D_5_-TCCTGCTCCT GC/TCCTGCTCCT GC-D_3_	41/42	SCSC
7	D_5_-GTCGGGTGTG AC/GTCGGGTGTG AC-D_3_	41/42	VGCD
8	D_5_-TTCATGTACG TG/TTCATGTACG TG-D_3_	41/42	FMYV
9	D_5_-TGCTGCCCAC GC/TGCTGCCCAC GC-D_3_	41/42	CCPR
10	D_5_-CCAGGCATTA AA/CCAGGCATTA AA-D_3_	41/42	PGIK
11	D_5_-GACCCATCCC AA/GACCCATCCC AA-D_3_	41/42	DPSQ
12	D_5_-GGAGGACGGC GG/GGAGGACGGC GG-D_3_	41/42	GGRR
13	D_5_-TCCGCCACTT GC/TCCGCCACTT GC-D_3_	41/42	SGTC
14	D_5_-GCCGCCTTTA GA/GGCGGCTTTA GA-D_3_	41/42	GGFR
15	D_5_-CTGAAGGACT AC/CTGAAGGACT AC-D_3_	41/42	LKDY
16	D_5_-AAAGATGACG AT/AAAGATGACG AT-D_3_	41/42	KDDD
17	D_5_-TCCCCAATTA TT/TCCCCAATTA TT-D_3_	41/42	SPII
18	D_5_-CCACCACCAT GC/CCACCACCAT GC-D_3_	41/42	PPPC
19	D_5_-CCACCATCCT GC/CCACCATCCT GC-D_3_	41/42	PPSC
20	D_5_-TGTGACTTCA TG/TGTGACTTCA TG-D_3_	41/42	CDFM
21	D_5_-CCACGCCCAG GC/CCACGCCCAG GC-D_3_	41/42	PRPG
22	D_5_-TCCCAAATTA TT/TCCCAAATTA TT-D_3_	41/42	SQII
23	D_5_-GGAGGCGGAG GC/GGAGGCGGAG GC-D_3_	41/42	GGGG

^a^For simplicity, the device sequences are defined and symbolized (D_5_  and D_3_). For the YLBS block shuffling, 5′-half and 3′-half oligonucleotides are hybridized at their stem sequences (i.e., D_5_ and D_3_) and then ligated (for detail, see [19]).

**Table 2:** CatE-activating peptides (at neutral pH) obtained from the primary library (A) and the secondary library (B) selections.

Name	Amino acid sequence (N→C)	Size (a.a.)	pI	Hydropathy (GRAVY^d^)	Activities (%)		K_D_
					A_dp_^a^	A_sy_^b^	
(A) Primary library selection							

P_1 _(p2006)	VGCDFMYV	8	3.8	1.3	118 ± 3.2	129 ± 10	N. A. (~10^−5^)^c^
P_2 _(p2005)	KGPPPCPC	8	8.2	−0.7	128 ± 20	110 ± 4	
P_3 _(p3006)	IEGRVGCDFMYVG	13	4.2	0.5	130 ± 10	130 ± 5	3.34 × 10^−7^

(B) Secondary library selection							

S_1 _(p4035)	SPIISHIVGCDPPSCG	16	5.3	0.5	160 ± 20	—	—
S_2 _(p4043)	PGIKPPPCIIIIG	13	8.6	1.07	180 ± 10	144.5 ± 11	N. A.^c^
S_3 _(p4038)	IGCEERSFPNIIIIIG	16	4.3	0.9	260 ± 10	168 ± 3	3.89 × 10^−7^

^a^A_dp _is the activity measured using the *in vitro *translated peptide.

^b^A_sy_ is the activity measured using the chemically synthesized peptide. For detail, see [18].

^c^Not accurate; SPR-measured repeatedly but the values obtained were not sufficiently reliable. A rough estimation is given in the parenthesis.

^d^Grand average of hydropathy value (source: http://www.bioinformatics.org/sms2/protein_gravy.html).

## References

[1] Southan C (2001). A genomic perspective on human proteases as drug targets. *Drug Discovery Today*.

[2] Turk B (2006). Targeting proteases: successes, failures and future prospects. *Nature Reviews Drug Discovery*.

[3] Putt KS, Chen GW, Pearson JM (2006). Small-molecule activation of procaspase-3 to caspase-3 as a personalized anticancer strategy. *Nature Chemical Biology*.

[4] Zorn JA, Wells JA (2010). Turning enzymes on with small molecules. *Nature Chemical Biology*.

[5] Ottmann C, Hauske P, Kaiser M (2010). Activation instead of inhibition: targeting proenzymes for small-molecule intervention. *ChemBioChem*.

[6] Hauske P, Ottmann C, Meltzer M, Ehrmann M, Kaiser M (2008). Allosteric regulation of proteases. *Chembiochem*.

[7] Wlodawer A, Li M, Gustchina A (2001). Inhibitor complexes of the Pseudomonas serine-carboxyl proteinase. *Biochemistry*.

[8] Redzynia I, Ljunggren A, Bujacz A, Abrahamson M, Jaskolski M, Bujacz G (2009). Crystal structure of the parasite inhibitor chagasin in complex with papain allows identification of structural requirements for broad reactivity and specificity determinants for target proteases. *FEBS Journal*.

[9] Kitamura K, Yoshida C, Kinoshita Y (2009). Development of systemic in vitro evolution and its application to generation of peptide-aptamer-based inhibitors of cathepsin E. *Journal of Molecular Biology*.

[10] Nemoto N, Miyamoto-Sato E, Husimi Y, Yanagawa H (1997). In vitro virus: bonding of mRNA bearing puromycin at the 3’-terminal end to the C-terminal end of its encoded protein on the ribosome in vitro. *FEBS Letters*.

[11] Roberts RW, Szostak JW (1997). RNA-peptide fusion for the in vitro selection of peptides and proteins. *Proceedings of the National Academy of Sciences of the United States of America*.

[12] Yamaguchi J, Naimuddin M, Biyani M (2009). cDNA display: a novel screening method for functional disulfide-rich peptides by solid-phase synthesis and stabilization of mRNA-protein fusions. *Nucleic Acids Research*.

[13] Naimuddin M, Kitamura K, Kinoshita Y (2007). Selection-by-function: efficient enrichment of cathepsin E inhibitors from a DNA library. *Journal of Molecular Recognition*.

[14] Kawakubo T, Okamoto K, Iwata JI (2007). Cathepsin E prevents tumor growth and metastasis by catalyzing the proteolytic release of soluble TRAIL from tumor cell surface. *Cancer Research*.

[15] DiPaola RS, Rinehart J, Nemunaitis J (2002). Characterization of a novel prostate-specific antigen-activated peptide-doxorubicin conjugate in patients with prostate cancer. *Journal of Clinical Oncology*.

[16] Athauda SBP, Takahashi K (2002). Distinct cleavage specificity of human cathepsin E at neutral pH with special preference for Arg-Arg bonds. *Protein and Peptide Letters*.

[17] Bromme D, Okamoto K (1995). Human cathepsin O2, a novel cysteine protease highly expressed in osteoclastomas and ovary molecular cloning, sequencing and tissue distribution. *Biological Chemistry Hoppe-Seyler*.

[18] Kitamura K, Yoshida C, Salimullah MD (2008). Rapid in vitro synthesis of pico-mole quantities of peptides. *Chemistry Letters*.

[19] Kitamura K, Kinoshita Y, Narasaki S, Nemoto N, Husimi Y, Nishigaki K (2003). Construction of block-shuffled libraries of DNA for evolutionary protein engineering: Y-ligation-based block shuffling. *Protein Engineering*.

[20] Alaimo PJ, Shogren-Knaak MA, Shokat KM (2001). Chemical genetic approaches for the elucidation of signaling pathways. *Current Opinion in Chemical Biology*.

[21] Huang R, Martinez-Ferrando I, Cole PA (2010). Enhanced interrogation: emerging strategies for cell signaling inhibition. *Nature Structural and Molecular Biology*.

[22] Goode DR, Totten RK, Heeres JT, Hergenrother PJ (2008). Identification of promiscuous small molecule activators in high-throughput enzyme activation screens. *Journal of Medicinal Chemistry*.

[23] Chothia C, Gough J, Vogel C, Teichmann SA (2003). Evolution of the protein repertoire. *Science*.

[24] Chothia C, Gough J (2009). Genomic and structural aspects of protein evolution. *Biochemical Journal*.

[25] Orengo CA, Thornton JM (2005). Protein families and their evolution—a structural perspective. *Annual Review of Biochemistry*.

